# Omega-3 fatty acid-containing parenteral nutrition in ICU patients: systematic review with meta-analysis and cost-effectiveness analysis

**DOI:** 10.1186/s13054-020-03356-w

**Published:** 2020-11-03

**Authors:** Lorenzo Pradelli, Stanislaw Klek, Konstantin Mayer, Abdul Jabbar Omar Alsaleh, Martin D. Rosenthal, Axel R. Heller, Maurizio Muscaritoli

**Affiliations:** 1 AdRes-Health Economics and Outcome Research, Via Vittorio Alfieri 17, 10121 Turin, Italy; 2 Department of General and Oncology Surgery With Intestinal Failure Unit, Stanley Dudrick’s Memorial Hospital, Tyniecka 15, 32-050 Skawina, Poland; 3 Medical Clinic 4, Pneumology and Sleep Medicine, ViDia Hospitals Karlsruhe, Südendstr. 32, 76137 Karlsruhe, Germany; 4 Division of Trauma and Acute Care Surgery, Department of Surgery, University of Florida College of Medicine, Gainesville, FL 32610-0019 USA; 5 Department of Anesthesiology and Intensive Care Medicine, University of Augsburg, Universitätsstraße 2, 86159 Augsburg, Germany; 6 Department of Clinical Medicine, Sapienza University of Rome, Piazzale Aldo Moro, 5, 00185 Rome, RM Italy; 7 Department of Economics, University of Bologna, Bologna, Italy

**Keywords:** Parenteral nutrition, Intensive care, Critically ill, Meta-analysis, Cost-effectiveness, Omega-3 fatty acid

## Abstract

**Background:**

Omega-3 (ω-3) fatty acid (FA)-containing parenteral nutrition (PN) is associated with significant improvements in patient outcomes compared with standard PN regimens without ω-3 FA lipid emulsions. Here, we evaluate the impact of ω-3 FA-containing PN versus standard PN on clinical outcomes and costs in adult intensive care unit (ICU) patients using a meta-analysis and subsequent cost-effectiveness analysis from the perspective of a hospital operating in five European countries (France, Germany, Italy, Spain, UK) and the US.

**Methods:**

We present a pharmacoeconomic simulation based on a systematic literature review with meta-analysis. Clinical outcomes and costs comparing ω-3 FA-containing PN with standard PN were evaluated in adult ICU patients eligible to receive PN covering at least 70% of their total energy requirements and in the subgroup of critically ill ICU patients (mean ICU stay > 48 h). The meta-analysis with the co-primary outcomes of infection rate and mortality rate was based on randomized controlled trial data retrieved via a systematic literature review; resulting efficacy data were subsequently employed in country-specific cost-effectiveness analyses.

**Results:**

In adult ICU patients, ω-3 FA-containing PN versus standard PN was associated with significant reductions in the relative risk (RR) of infection (RR 0.62; 95% CI 0.45, 0.86; *p* = 0.004), hospital length of stay (HLOS) (− 3.05 days; 95% CI − 5.03, − 1.07; *p* = 0.003) and ICU length of stay (LOS) (− 1.89 days; 95% CI − 3.33, − 0.45; *p* = 0.01). In critically ill ICU patients, ω-3 FA-containing PN was associated with similar reductions in infection rates (RR 0.65; 95% CI 0.46, 0.94; *p* = 0.02), HLOS (− 3.98 days; 95% CI − 6.90, − 1.06; *p* = 0.008) and ICU LOS (− 2.14 days; 95% CI − 3.89, − 0.40; *p* = 0.02). Overall hospital episode costs were reduced in all six countries using ω-3 FA-containing PN compared to standard PN, ranging from €-3156 ± 1404 in Spain to €-9586 ± 4157 in the US.

**Conclusion:**

These analyses demonstrate that ω-3 FA-containing PN is associated with statistically and clinically significant improvement in patient outcomes. Its use is also predicted to yield cost savings compared to standard PN, rendering ω-3 FA-containing PN an attractive cost-saving alternative across different health care systems.

**Study registration:**

PROSPERO CRD42019129311.

## Background

Parenteral nutrition (PN) is offered to patients if oral or enteral nutrition is impossible, insufficient, or contraindicated. One integral component of PN is lipids, as they provide an energy-dense source of calories, essential fatty acids (FA) and the building blocks for cell membranes [1, 2]. A number of different sources for the lipid emulsions used in PN have been developed over time, the first of which was soybean oil [1]. One potential disadvantage of using emulsions containing soybean oil as the sole lipid source is the relatively high omega-6 (ω-6) polyunsaturated fatty acid (PUFA) content: Over 50% of the FAs in soybean oil are comprised of linoleic acids [1, 3]. Following concerns that ω-6 PUFAs might have pro-inflammatory and immunosuppressive properties, mixtures of different lipid sources were developed to partially replace linoleic acid and α-linolenic acid in PN with medium-chain triglycerides, monounsaturated fatty acids from olive oil and/or eicosapentaenoic acid (EPA) and docosahexaenoic acid (DHA) from fish oil [1, 4]. Omega-3 (ω-3) FAs derived from fish oil may offer clinical benefits across a wide spectrum of patients, including patients in an intensive care unit (ICU), due to their effects on anti-inflammatory, immunomodulatory, and pro-resolution pathways [1, 3, 4].

Meta-analyses have demonstrated the positive impact of ω-3 FA-containing PN in comparison with standard PN (without ω-3 FA supplementation) on critically ill adults, with statistically significant reductions in hospital length of stay (HLOS) [5–14], ICU length of stay (LOS) [8, 15], duration of mechanical ventilation [15, 16] and decreased infection rates [7–9, 11–13, 17]. The risk of infection increases with longer HLOS [18] and a shorter ICU LOS reduces general deconditioning due to prolonged bed rest, sedation and immobilization, which negatively impact patient quality of life [19].

Patients treated in an ICU setting are a specific group within the overall population of hospitalized patients. ICU patients tend to have more severe medical conditions and the treatment costs for ICU patients are generally higher than those for other patients in a routine hospital setting [20]. Critical illnesses can trigger acute metabolic changes leading to hypercatabolism [21]. Consequently, patients are at high risk of developing energy and protein deficits during an ICU stay, resulting in loss of lean body mass and ICU-acquired weakness [22–30]. In critically ill patients, hypercatabolism is associated with poor clinical outcomes, which can be counteracted by appropriate feeding [22, 23]. Adequate protein and calorie delivery are key for the maintenance and generation of muscle mass, strength and function [28, 31]. In ICU patients, hypocaloric or hypercaloric energy intake over a prolonged period increases the risk of negative clinical outcomes and results in higher infection and mortality rates as well as a longer duration of mechanical ventilation [22, 23, 32–36].

Meta-analyses and systematic reviews are important tools for clinicians and patients to inform treatment decisions via the evaluation of health care intervention risks/benefits and can provide a starting point for the development of clinical practice guidelines [37]. Cost-effectiveness analyses compare new interventions with current clinical practice in terms of costs and benefits; both of these outcomes may influence treatment decisions as well as guideline development [38]. A recent meta-analysis and the corresponding cost-effectiveness analysis in hospitalized patients treated with PN (i.e., patients in a general ward and/or in an ICU) confirmed that ω-3 FA-containing PN is not only associated with clinical benefits compared to standard PN without ω-3 fatty acids but also with concurrent cost savings [8, 39].

While these results suggest clinical and economic benefits in the hospitalized patient population as a whole, it was not designed to specifically inform the effects on the ICU population, which was a prespecified subgroup analyzed only, as appropriate, to investigate sources of heterogeneity in the overall estimates. We could therefore not evaluate if the overall results would also apply to the ICU population, and even less in the subgroup of critically ill (as opposed to patients transiting in the ICU for post-surgical weaning and monitoring). In this analysis on the population of ICU patients, we determine the influence of ω-3 FA-containing PN versus standard PN on clinical outcomes and simulate associated treatment costs across five European countries (France, Germany, Italy, Spain and the UK) and the US.

## Methods

The present study is comprised of a meta-analysis and a cost-effectiveness analysis in order to evaluate the impact of omega-3 fatty acid-containing PN versus standard PN (not supplemented with ω-3 FA) on clinical outcomes and costs in adult ICU patients.

### Meta-analysis

#### Overview

The protocol for this study was published prospectively (PROSPERO CRD42019129311). In summary, the meta-analysis included the following steps: (1) definition of eligibility criteria, (2) identification of databases and search strategy, (3) structured literature search to identify publications, followed progressively by study selection based on title, abstract, and full text, and (4) data extraction and synthesis of the results.

#### Patient population

This analysis included data from hospitalized adult ICU patients (as defined by the authors of each study) who were eligible to receive PN to cover at least 70% of their total energy requirements. Two groups of ICU-patients were investigated: (1) all patients treated in an ICU setting (as defined by the authors of each study), and (2) a subgroup of ICU patients comprising “critically ill” patients (as defined by the authors of each study or with a mean ICU stay > 48 h). Non-target populations (i.e., pediatric or neonatal patients, general ward) or enteral nutrition studies were excluded.

#### Outcomes

The primary and co-primary outcomes were infection rate (any nosocomial infection) and mortality rate (30-day mortality: any death occurring up to 30 days after receiving at least one dose of study treatment), respectively. Secondary outcomes included HLOS, ICU LOS, sepsis rate and length of mechanical ventilation. Other outcomes included transfused blood units and oxygenation index, the fatty-acid composition of plasma phospholipids and the lipid profile (α-tocopherol, EPA, DHA, arachidonic acid, plasma triglycerides), markers of inflammation and antioxidant status (interleukin-6, leukotriene [LT] B5, LTB4, LTB5:LTB4 ratio, C-reactive protein, tumor necrosis factor [TNF]-α), and routine laboratory parameters (lactate; urea; serum creatinine; creatinine clearance; platelets; prothrombin time; partial thromboplastin time [PTT]; international normalized ratio; bleeding time; liver enzymes aspartate [AST] and alanine aminotransferase [ALT], and γ–glutamyl transferase [GGT]; and total bilirubin).

#### Statistical verification

Trial sequential analysis (TSA) was used to explore whether the cumulative evidence was sufficiently robust to reach conclusive results; i.e., whether the pooled analyses were adequately powered to reliably evaluate the treatment effect on outcomes. A TSA was performed for all primary and secondary outcomes with a statistically significant pooled effect.

#### Meta-bias

To determine the presence of reporting biases, we determined whether a protocol for the respective randomized clinical trial was published before its conduct. Studies with a published protocol were evaluated for selective reporting of outcomes (outcome reporting bias). Reporting biases were further explored via funnel plots if ≥ 10 studies were available. To evaluate the confidence in the cumulative estimates for all statistically significant outcomes, the *Grading of Recommendations Assessment, Development and Evaluation* (GRADE) working group methodology was applied using GRADEpro v.3.6.1 [40].

### Pharmacoeconomic analysis

#### Overview

Six separate cost-effectiveness models comparing ω-3 FA-containing PN with standard PN (without ω-3 FA supplementation) in ICU patients were developed and simulated for hospitals in France, Germany, Italy, Spain, the UK and the US. The model generation included the following steps: (1) conceptualization of a logical structure for both patient cohorts [all ICU patients and critically ill ICU patients]; (2) identification of country-specific outcomes for patients receiving standard PN in both cohorts; (3) identification of country-specific sources for drug acquisition and hospital service costs; (4) simulation of country-specific outcomes for patients receiving ω-3 FA-containing PN by applying the results of the meta-analysis; (5) calculation of the country-specific total cost per simulated patient; (6) analysis of the result’s sensitivity to input parameter uncertainty via deterministic and probabilistic sensitivity analyses (PSA). The models were based on a probabilistic discrete event simulation technique and developed in Excel (Microsoft Corporation, Redmond, WA, USA). Simulations were run over 10,000 iterations, with each iteration representing one patient. Costs were modeled in the currencies of each country (pound sterling [GBP] in the UK and US dollars [USD] in the US), but converted to euros (EUR) using the average exchange rate of January 2020 to facilitate comparability. The conversion rates were as follows: GBP-EUR 1.1759 and USD-EUR 0.9005.

#### Patient population

All models included two treatment arms (ω-3 FA-containing PN and standard PN), with each patient passing simultaneously through both; i.e., both simulated alternatives run on the same cohort. Infection episodes, discharge from hospital, and death were evaluated. The latter two parameters defined the end of the patient pathway, whereas the former only affected treatment costs.

Outcomes of the patient cohort were simulated according to the PN treatment regimen they received and compared: ω-3 FA-containing PN versus standard PN (Additional file 1: Figure S1).

Country-specific patient outcomes for the standard PN receiving group relevant for the simulation (lengths of stay, death and infection rates) were retrieved in published sources (Additional file 1: Table S1) and an appropriate distribution fitted to represent them in the model using the method of moments. Outcomes for the ω-3 FA-containing PN group are simulated after applying the relative efficacy estimates from the meta-analyses to the outcomes of the standard PN group.

#### Model cost inputs

The input parameters for ICU-patients in six countries (France, Germany, Italy, Spain, UK and US), such as daily costs, costs per infection, and costs for PN treatment, were extracted from published sources and are displayed in Additional file 1: Table S1.

For the European countries, the daily PN costs were based on current market shares and prices as well as the daily number of PN bags required per patient. For US daily cost estimations, daily lipid requirements were modeled based on patient age distribution [41] and patient weight in gender- and age-specific groups [42]. Costs of lipid emulsions were based on the lowest price for standard PN while using manufacturer prices for ω-3 FA-containing PN.

#### Sensitivity analyses

Probabilistic and deterministic sensitivity testing approaches were used to determine the influence of model parameters on calculated estimates. A probabilistic sensitivity analysis (PSA) determines the effect of global parameter uncertainty on estimated costs. PSAs were performed by drawing parameter values from their respective probability distributions, thus creating 1000 unique sets of parameter combinations. If data on uncertainty were missing, a 20% standard deviation of the mean value with an appropriate probability distribution was used.

In deterministic sensitivity analyses, simulations were repeated while varying parameter values to their lower and upper confidence interval limits and keeping the remaining parameter values constant. In cases of unavailable confidence intervals, the lower and upper 95% confidence interval limits of the distribution used in the PSA were assumed as parameter values.

## Results

### Systematic literature review and meta-analysis

#### Study selection and characteristics

Four thousand and three publications were initially identified via database searches. Of these, 69 publications remained relevant after the titles and abstracts were screened by three independent reviewers. Following the evaluation of the full-text articles and the exclusion of publications that did not meet eligibility criteria, this number was further reduced to 24 studies (Additional file 1: Figure S2). Therefore, our meta-analysis of ω-3 FA-containing PN versus standard PN includes 24 randomized controlled trials with a total of 1421 patients receiving PN in an ICU setting (Additional file 1: Table S2).

#### Clinical outcomes

**Fig. 1:**
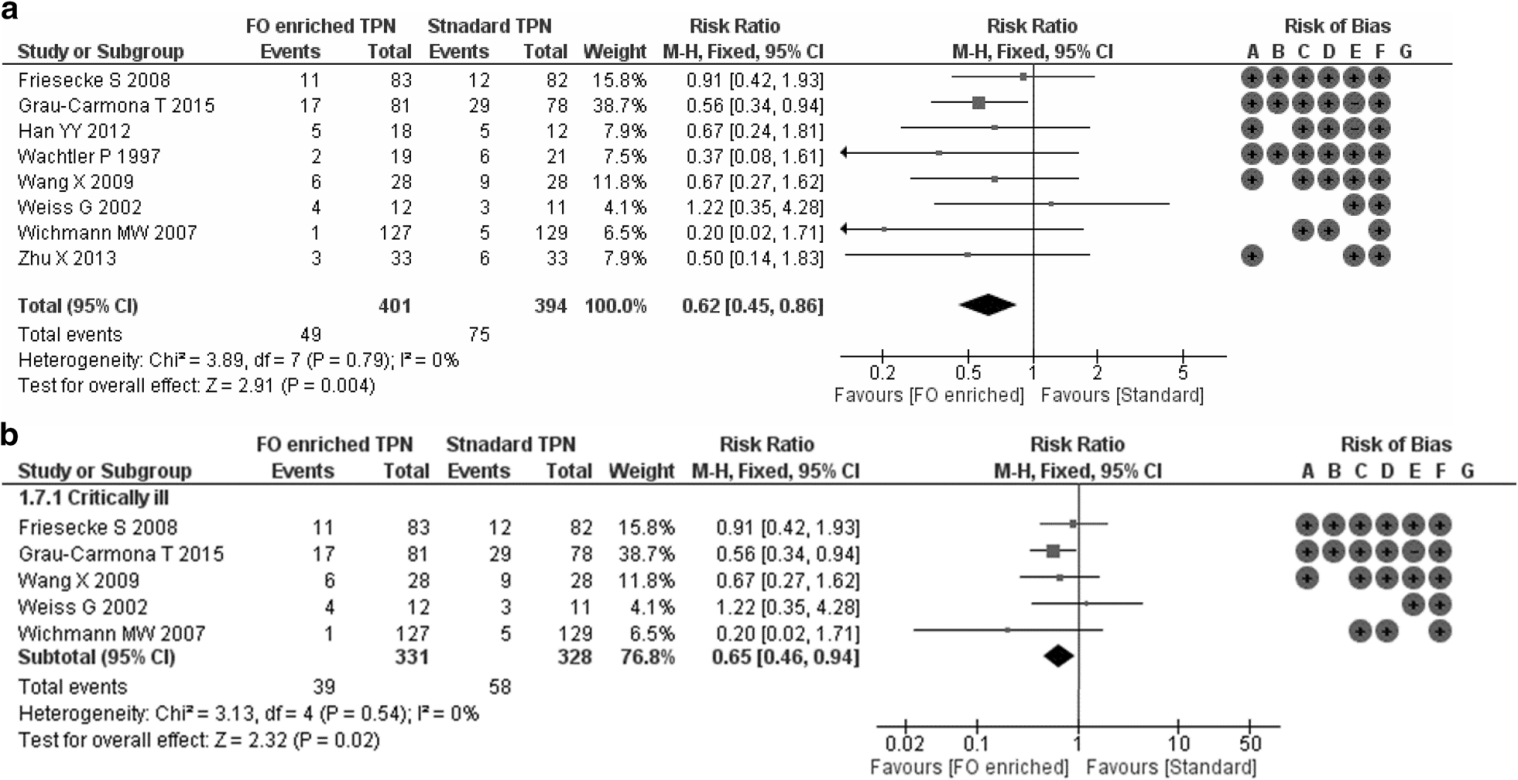
Infection rates in **a** all ICU patients and **b** critically ill ICU patients. Risk of bias legend: +: high risk; −; low risk; blank: risk unclear. A: random sequence generation (selection bias); B: allocation concealment (selection bias); C: blinding of participants and personnel (performance bias); D: blinding outcome data (attrition bias); E: incomplete outcome data (attrition bias); F: selective reporting (reporting bias); G: other bias. CI, confidence interval; FA, fatty acid; ICU, intensive care unit; ω-3, omega-3; M–H, Mantel–Haenszel study weighting; PN, parenteral nutrition

**Fig. 2:**
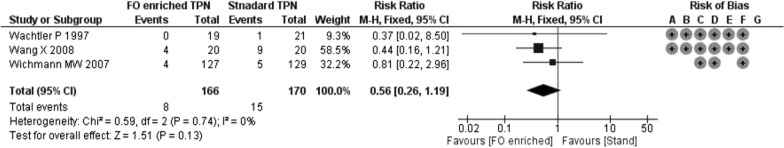
Sepsis in ICU patients. Risk of bias legend: +: high risk; −; low risk; blank: risk unclear. A: random sequence generation (selection bias); B: allocation concealment (selection bias); C: blinding of participants and personnel (performance bias); D: blinding outcome data (attrition bias); E: incomplete outcome data (attrition bias); F: selective reporting (reporting bias); G: other bias. CI, confidence interval; FA, fatty acid; ICU, intensive care unit; ω-3, omega-3; M–H, Mantel–Haenszel study weighting; PN, parenteral nutrition

**Fig. 3:**
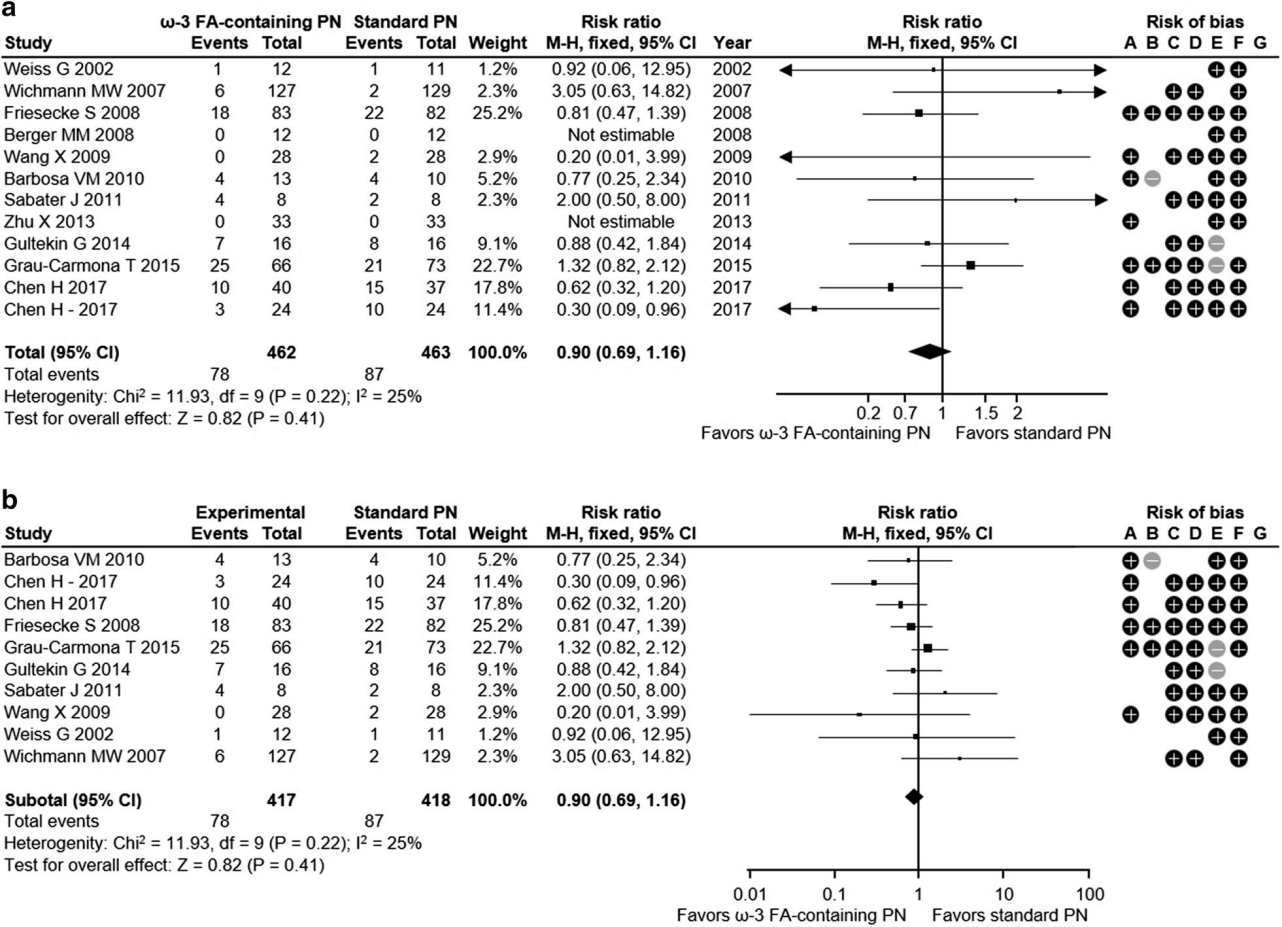
Thirty-day mortality in **a** all ICU patients and **b** critically ill ICU patients. Risk of bias legend: +: high risk; −; low risk; blank: risk unclear. A: random sequence generation (selection bias); B: allocation concealment (selection bias); C: blinding of participants and personnel (performance bias); D: blinding outcome data (attrition bias); E: incomplete outcome data (attrition bias); F: selective reporting (reporting bias); G: other bias. CI, confidence interval; FA, fatty acid; ICU, intensive care unit; ω-3, omega-3; M–H, Mantel–Haenszel study weighting; PN, parenteral nutrition

**Fig. 4:**
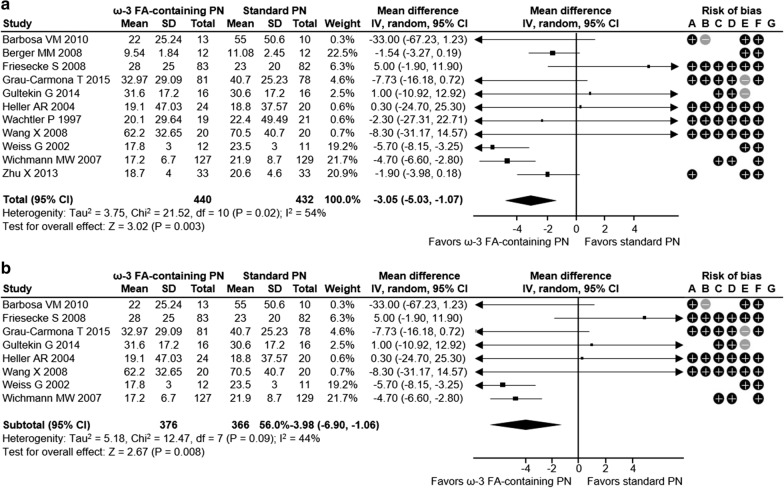
HLOS in **a** all ICU patients and **b** critically ill ICU patients. Risk of bias legend: +: high risk; −; low risk; blank: risk unclear. A: random sequence generation (selection bias); B: allocation concealment (selection bias); C: blinding of participants and personnel (performance bias); D: blinding outcome data (attrition bias); E: incomplete outcome data (attrition bias); F: selective reporting (reporting bias); G: other bias. CI, confidence interval; FA, fatty acid; HLOS, hospital length of stay; ICU, intensive care unit; ω-3, omega-3; M–H, Mantel–Haenszel study weighting; PN, parenteral nutrition

**Fig. 5:**
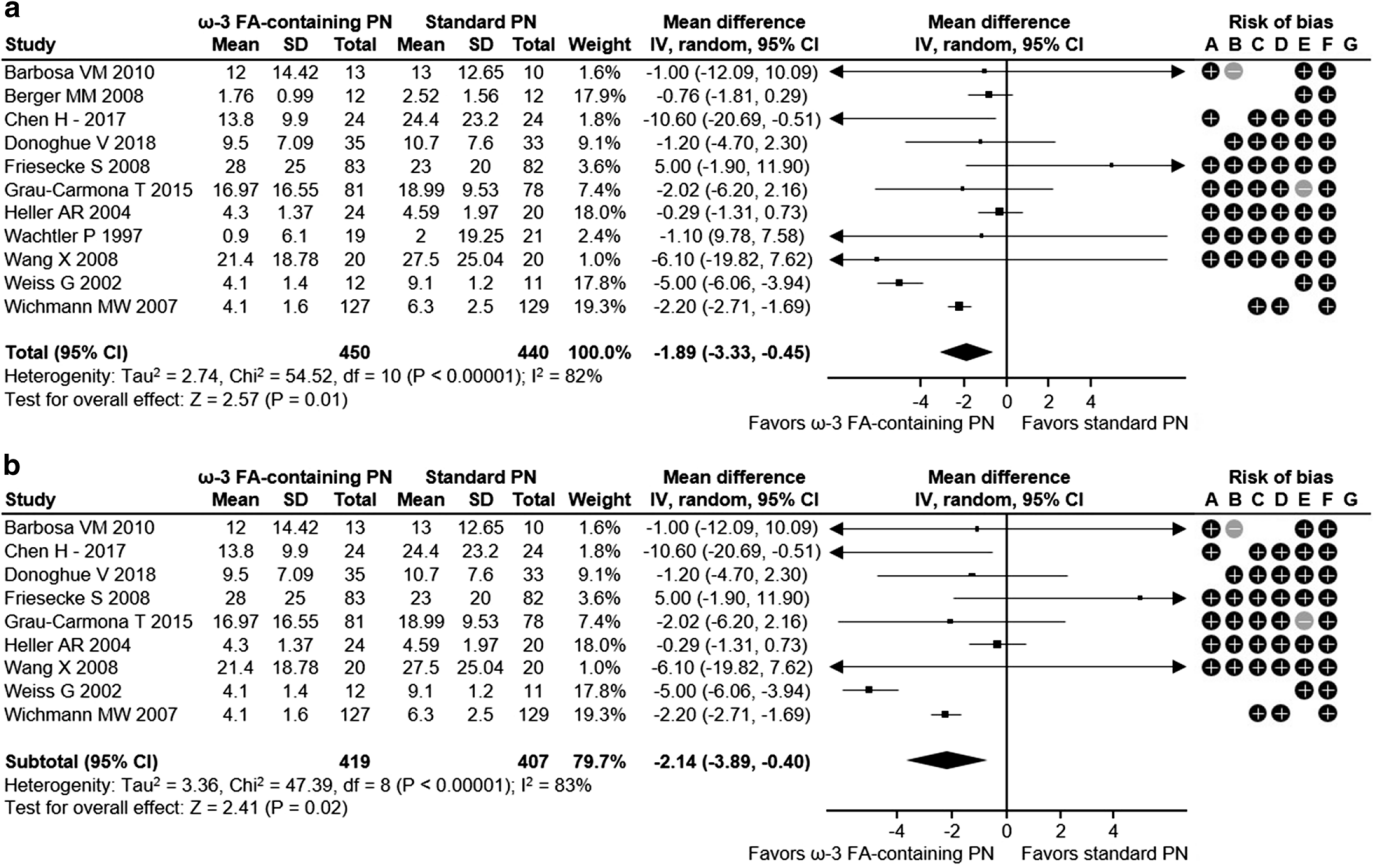
ICU LOS in **a** all ICU patients and **b** critically ill ICU patients. Risk of bias legend: +: high risk; −; low risk; blank: risk unclear. A: random sequence generation (selection bias); B: allocation concealment (selection bias); C: blinding of participants and personnel (performance bias); D: blinding outcome data (attrition bias); E: incomplete outcome data (attrition bias); F: selective reporting (reporting bias); G: other bias. CI, confidence interval; FA, fatty acid; ICU, intensive care unit; ω-3, omega-3; M–H, Mantel–Haenszel study weighting; PN, parenteral nutrition

**Fig. 6:**
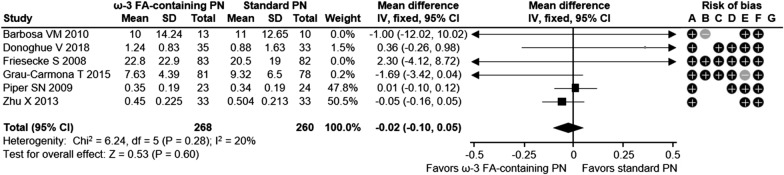
Length of mechanical ventilation in ICU patients. Risk of bias legend: +: high risk; −; low risk; blank: risk unclear. A: random sequence generation (selection bias); B: allocation concealment (selection bias); C: blinding of participants and personnel (performance bias); D: blinding outcome data (attrition bias); E: incomplete outcome data (attrition bias); F: selective reporting (reporting bias); G: other bias. CI, confidence interval; FA, fatty acid; ICU, intensive care unit; ω-3, omega-3; M–H, Mantel–Haenszel study weighting; PN, parenteral nutrition

Overall, this meta-analysis showed a positive impact of omega-3 FA-containing PN on clinical outcomes for ICU patients. The relative risk for infection was reduced significantly by 38% with ω-3 FA-containing PN versus standard PN in ICU patients across 8 studies with 795 patients (relative risk [RR] 0.62; 95% confidence interval [CI] 0.45, 0.86; *p* = 0.004) (Fig. 1a). Similarly, critically ill ICU patients had a 35% RR reduction for infection with ω-3 FA-containing PN (5 studies with 659 patients; RR 0.65; 95% CI 0.46, 0.94; *p* = 0.02) (Fig. 1b). There was a not significant trend towards a decreasing incidence of sepsis with ω-3 FA-containing PN (RR 0.56; 95% CI 0.26, 1.19; *p* = 0.13) in 3 studies with 336 ICU patients (Fig. 2). Sepsis was not evaluated for the subgroup of critically ill patients, as the minimum observation requirement was not met. ω-3 FA-containing PN was associated with a non-significant 10% relative risk reduction in 30-day mortality in all ICU patients and the subgroup of critically ill ICU patients across 12 studies with 925 patients (RR 0.90; 95% CI 0.69, 1.16; *p* = 0.41) and 10 studies with 835 patients (RR 0.90; 95% CI 0.69, 1.16; *p* = 0.41), respectively (Fig. 3a, b). HLOS was reported in 11 studies with 872 ICU patients and 8 studies with 742 critically ill ICU patients: both patient groups exhibited significant reductions in mean HLOS with ω-3 FA-containing PN of − 3.05 days (95% CI − 5.03, − 1.07; *p* = 0.003) and − 3.98 days (95% CI − 6.90, − 1.06; *p* = 0.008), respectively (Fig. 4a, b). ICU LOS was also significantly reduced with ω-3 FA-containing PN versus standard PN in all ICU patients and critically ill ICU patients: 11 studies (890 patients) reported ICU LOS in the general ICU patient population, with a mean reduction of − 1.89 days (95% CI − 3.33, − 0.45; *p* = 0.01) (Fig. 5a) and 9 studies with 826 patients reported a mean ICU LOS reduction of − 2.14 days (95% CI − 3.89, − 0.40; *p* = 0.02) in critically ill ICU patients (Fig. 5b). Length of mechanical ventilation was reported in 6 studies (528 patients): Compared with standard PN, ω-3 FA-containing PN was associated with a non-significant reduction of − 0.02 days in length of mechanical ventilation (95% CI − 0.10, 0.05; *p* = 0.60) (Fig. 6). The relevance of statistically significant outcomes was verified with TSAs: all significant clinical outcomes showed adequate statistical power, meaning the results can be considered conclusive (Additional file 1: Figure S3). Hence, the beneficial effects of a lower infection rate, shorter HLOS as well as ICU LOS are indeed a consequence of the application of an ω-3 FA-containing PN solution.

#### Laboratory outcomes

Patients receiving ω-3 FA-containing PN also experienced a significant benefit regarding several laboratory parameters compared with patients receiving standard PN: A liver parameter (ALT), inflammatory markers (interleukin [IL]-6, TNF- α, LTB4:LTB5 ratio), the lipid profile and composition (EPA and DHA) as well as the concentration of liposoluble vitamins (α-tocopherol) all significantly improved with the administration of ω-3 FA-containing PN (Additional file 1: Table S3).

#### Confidence in cumulative estimates and meta-bias

Confidence in the cumulative estimates of analyzed clinical outcomes, according to the GRADE evaluation, was high for the clinical outcome infection rate and moderate for HLOS and ICU LOS (Additional file 1: Table S4). Regarding laboratory parameters, confidence was either high or moderate. Funnel plots were used to evaluate potential meta-biases (reporting bias) in clinical outcome analyses. The Funnel plots appeared symmetrical for 30-day mortality and HLOS and slightly asymmetrical for infection rate, ICU LOS and mechanical ventilation; however, no evidence for significant bias on the weighted regression was found using both Begg’s and Egger’s tests (Additional file 1: Figure S4).

### Simulation and cost-effectiveness

#### Pharmacoeconomic analysis

Additional file 1: Table S1 summarizes all input parameters for ω-3 FA-containing PN and standard PN. The simulation results revealed that mean HLOS and mean incidence of infection with ω-3 FA-containing PN and standard PN varied widely between countries and treatment groups (Table 1). The cost of PN acquisition was higher with ω-3 FA-containing PN in all countries analyzed except France (Table 2). Nevertheless, all five European and the US ICU settings investigated, the increased effectiveness of ω-3 FA-containing PN correlated with a decrease in mean cost per adult patient. Total cost reductions amounted to €-3652 ± 783 in France, €-4813 ± 1011 in Germany, €-3342 ± 1254 in Italy, €-3156 ± 1404 in Spain, €-5546 ± 436 (£-4986 ± 392) in the UK and €-9586 ± 4157 ($-10,672 ± 4628) in the US. In all six countries, expenses for infections and HLOS were lower with ω-3 FA-containing PN versus standard PN, with the US exhibiting the largest savings for both (infection: €-850 ± 5055/$-947 ± 4628; HLOS: €-8856 ± 0/$-9869 ± 0). Spain and the UK had lowest cost savings with ω-3 FA-containing PN versus standard PN regarding HLOS (€-2928 ± 0) and infections (€-65 ± 428/£-58 ± 385), respectively.

**Table 1:** Efficacy estimates: HLOS and infection with ω-3 FA-containing PN and standard PN

Mean efficacy		HLOS (days)	Incidence of infections (%)
France	ω-3 FA-containing PN	28.5	30
	Standard PN	31.5	47
Germany	ω-3 FA-containing PN	29.4	12
	Standard PN	32.5	19
Italy	ω-3 FA-containing PN	34.2	29
	Standard PN	37.3	45
Spain	ω-3 FA-containing PN	43.3	29
	Standard PN	46.3	46
UK	ω-3FA-containing PN	16.9	12
	Standard PN	19.9	20
US	ω-3 FA-containing PN	17.32	22
	Standard PN	20.37	35

FA, fatty acid; HLOS, hospital length of stay; ω-3, omega-3; PN, parenteral nutrition

**Table 2:** Modeled costs for PN, infections and HLOS with ω-3 FA-containing PN and standard PN

Mean costs ± SD (€)		PN	Infection	HLOS	Total
France	ω-3 FA-containing PN	293 ± 152	344 ± 530	31,869 ± 20,825	32,507 ± 20,866
	Standard PN	335 ± 171	543 ± 580	35,282 ± 20,825	36,159 ± 20,860
	∆	− 42 ± 35	− 199 ± 782	− 3413 ± 0	− **3652 ± 783**
Germany	ω-3 FA-containing PN	880 ± 581	230 ± 639	45,783 ± 29,324	46,893 ± 29,375
	Standard PN	804 ± 553	373 ± 781	50,528 ± 29,324	51,706 ± 29,347
	∆	76 ± 63	− 143 ± 1010	− 4745 ± 0	− **4813 ± 1011**
Italy	ω-3 FA-containing PN	918 ± 557	531 ± 838	37,928 ± 31,886	39,377 ± 31,934
	Standard PN	579 ± 362	833 ± 923	41,308 ± 31,886	42,719 ± 31,915
	∆	339 ± 202	− 302 ± 1242	− 3380 ± 0	− **3342 ± 1254**
Spain	ω-3 FA-containing PN	345 ± 243	612 ± 949	41,543 ± 22,990	42,500 ± 23,056
	Standard PN	222 ± 157	963 ± 1039	44,471 ± 22,990	45,656 ± 23,036
	∆	123 ± 88	− 351 ± 1402	− 2928 ± 0	− **3156 ± 1404**
UK^a^	ω-3 FA-containing PN	476 ± 220	101 ± 271	30,359 ± 35,183	30,936 ± 35,232
	Standard PN	467 ± 216	166 ± 330	35,850 ± 35,183	36,481 ± 35,201
	∆	9 ± 75	− 65 ± 428	− 5491 ± 0	− **5546 ± 436**
US^a^	ω-3 FA-containing PN	180 ± 111	1437 ± 2731	50,352 ± 45,650	51,970 ± 45,773
	Standard PN	51 ± 31	2288 ± 3150	59,217 ± 45,650	61,556 ± 45,751
	∆	128 ± 82	− 850 ± 5055	− 8865 ± 0	− **9586 ± 4157**

Bold values indicate the expected difference in total hospital costs per PN patient

EUR, euro; GBP, pound sterling; FA, fatty acid; HLOS, hospital length of stay; ω-3, omega-3; PN, parenteral nutrition; SD, standard deviation; USD, US dollar

^a^GBP and USD converted to EUR using the average exchange rates of January 2020: GBP-EUR: 1.1759; USD-EUR: 0.9005

#### Sensitivity analysis

**Fig. 7:**
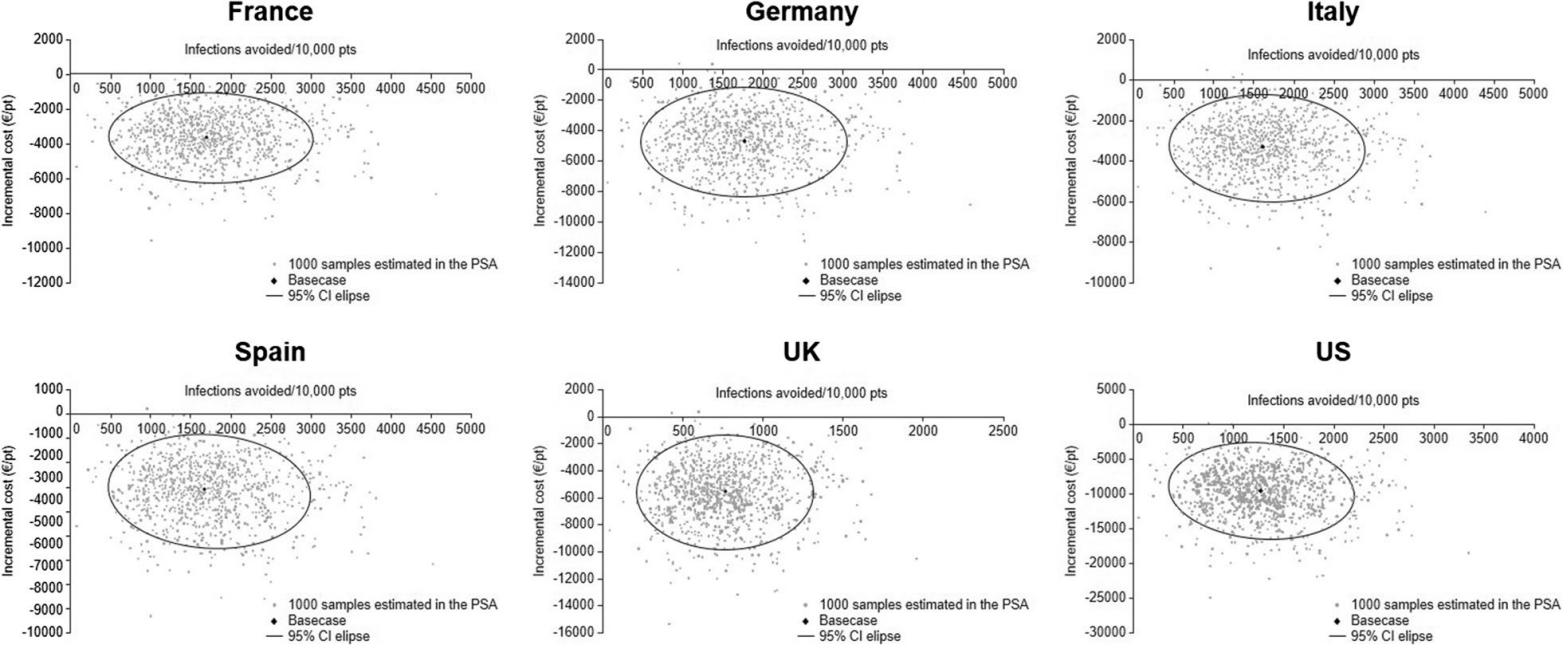
Scatterplots of 1000 ICER estimates in country-specific probabilistic sensitivity analyses. CI, confidence interval; ICER, incremental cost-effectiveness ratio; PSA, probabilistic sensitivity analysis; pt, patient

The stability and robustness of the cost-effectiveness analyses were confirmed with sensitivity analyses. Results for the PSAs are depicted in Fig. 7: For all six countries analyzed, 99.7–99.9% of the 1000 incremental cost-effectiveness ratio estimates, each using a different set of randomly drawn parameter values, verified that ω-3 FA-containing PN was cost-saving when compared with standard PN. According to our analysis, in order not to reduce costs versus standard PN in adult patients, ω-3 FA-containing PN would need to have a daily cost equal to €343.85 in France, €843.04 in Germany, €712.52 in Italy, €226.91 in Spain €925.91/£832.47 in the UK, and US$1826.62 in the US. Deterministic sensitivity analyses, which rank the influence of key parameters on cost savings per patient, showed that across all six countries and both PN treatment regimens the mean difference of HLOS was the most influential factor, followed by the daily costs for critically ill patients (Additional file 1: Figure S4).

## Discussion

ω-3 FA-containing PN has been associated with significantly improved patient outcomes across a wide range of studies [5–7, 9–11, 43–45] and our recently published analyses on hospitalized patients concluded that ω-3 FA-containing PN significantly improves clinical outcomes [8] while simultaneously leading to total cost savings in five European countries (France, Germany, Italy, Spain and the UK) and in the US when compared with standard PN [39]. The presented updated model indicates that the situation in ICU patients mirrors that of the overall hospital population: ω-3 FA-containing PN compared to standard PN is associated with significantly improved clinical outcomes and with concurrent cost reductions. This suggests that, from a hospital’s point of view, ω-3 FA-containing PN is likely a dominant alternative to standard PN for ICU patients. The higher acquisition cost for ω-3 FA-containing PN in comparison to standard PN is offset by lower overall expenses due to shorter HLOS, ICU LOS and lower infection rates; demonstrating that overall costs rather than nutrition cost should be considered in treatment decisions. The impact of ω-3 FA-containing PN on costs might be even greater when taking into consideration that at least 1 in 4 critically ill patients suffer from ICU-acquired weakness, a condition caused by hypercatabolism that results in severe loss of muscle mass, which is associated with poorer clinical outcomes such as higher hospital mortality, longer duration of mechanical ventilation and impaired long-term physical function [25–30]. Consequently, ICU patients often require subsequent rehabilitation, are unable to return to work or require nursing home care [46, 47].

Similar to the meta-analysis of all hospitalized patients [8], ω-3 FA-containing PN was associated with a better efficacy than standard PN in ICU patients. Compared to the overall population of hospitalized patients, ICU patients had a longer HLOS and a higher incidence of infection, which was to be expected in a more ill patient population. The reductions with ω-3 FA-containing PN regarding infection, sepsis and 30-day mortality were slightly less pronounced in ICU patients than in the general hospitalized patient population. However, there was an even greater reduction in HLOS among ICU patients with ω-3 FA-containing PN than previously reported for all hospitalized patients (ICU patients: − 3.05 days [95% CI − 5.03; − 1.07]; hospitalized patients: − 2.14 days [95% CI − 2.93; − 1.36]). Similarly, the subgroup of critically ill ICU patients experienced a greater treatment benefit with ω-3 FA-containing PN in terms of reduced ICU LOS compared with the general population of ICU patients or hospitalized patients (critically ill ICU patients: − 2.14 days [95% CI − 3.89; − 0.40]; all ICU patients: − 1.89 days [95% CI − 3.33; − 0.45]; hospitalized patients: − 1.95 days [95% CI − 3.49; − 0.42]). This suggests that ω-3 FA-containing PN may be particularly suited to benefit ICU patients who are critically ill (as defined by the authors of published data or with a mean ICU LOS > 48 h).

Despite the higher acquisition cost of ω-3 FA-containing PN, supplementation of ω-3 FA was associated with total cost savings in ICU patients. This cost reduction was even more significant in the ICU setting than in the general hospital setting for all five European countries analyzed [39]. The greater impact of ω-3 FA-containing PN on costs was likely due to the fact that daily costs for ICU patients are generally higher than for other hospitalized patients [20]. In European countries, such as Germany, Italy, Spain and the UK, ICU costs vary to some extent, with the UK having the most expensive ICU care [48]. Daily ICU costs in the US, however, are significantly higher [49], suggesting that reduction in ICU LOS will particularly benefit countries with high daily costs per ICU patient. Due to the increased severity of illnesses in hospitalized patients and an aging population, ICU costs have increased over the past decades and are expected to rise even further in the future [50, 51]. In this context, reducing overall costs may become an increasingly important outcome in the future.

The present meta-analysis is only partially comparable with the one previously conducted by Manzanares et al. [12], as it differs in the type of nutrition considered (we focus on total parenteral nutrition) and uses a different definition of critically ill (i.e., at least 5% mortality in the control group); it is nevertheless reassuring that the results of the two approaches compare well, with almost identical estimates of the effect on infection rate.

As both the meta-analysis and pharmacoeconomic analysis relied on the availability of published data regarding treatment effect and overall costs, one limitation of this study was dated references. Additionally, the population of ICU patients reported is rather heterogeneous and dependent on the medical condition, which results in great variation regarding hospital and ICU LOS. However, sensitivity analyses have shown that the overall direction of the results, i.e., better clinical outcomes at lower health care cost, is robust to reasonable variations of the context.

The accumulation of evidence regarding the improvement of clinical outcomes with ω-3 FA-containing PN in comparison to standard PN [5–7, 9–11, 43–45] may contribute to evidence-based treatment decisions and future clinical practice guidelines. The simultaneous cost savings with PN containing ω-3 FA, as shown here for ICU patients and previously for hospitalized patients in general [39], may require consideration with respect to the best standard of care in PN.

## Conclusions

This meta-analysis is the largest to date investigating the effects of different intravenous PN regimens on clinically important outcomes in the ICU. Here, we show that ω-3 FA-containing PN is associated with a positive impact on clinical outcomes for ICU patients, such as reductions in infection rates and the duration of hospital and ICU stay. A pharmacoeconomic simulation revealed that ω-3 FA-containing PN is likely a dominant alternative to standard PN from a hospital’s point of view for all of the five European countries analyzed (France, Germany, Italy, Spain and the UK) as well as for the US. Despite higher acquisition costs for ω-3 FA-containing PN, its superior efficacy makes it a cost-saving alternative. Here, we confirm the positive impact of ω-3 FA-containing PN demonstrated for hospitalized patients [8] in both the general population of ICU patients and in the subgroup of critically ill ICU patients.

## Supplementary information

**Additional file 1:** Complete input parameters, included studies details and additional results and analyses.

## Data Availability

All input parameters of the meta-analysis and pharmacoeconomic analysis were based on published literature.

## Abbreviations

ALT: Alanine aminotransferase
AST: Aspartate aminotransferase
CI: Confidence interval
DHA: Docosahexaenoic acid
EPA: Eicosapentaenoic acid
EUR: Euros
FA: Fatty acid
GBP: Pound sterling
GGT: γ-Glutamyl transferase
HLOS: Hospital length of stay
ICU: Intensive care unit
IL: Interleukin
LOS: Length of stay
LT: Leukotriene
PN: Parenteral nutrition
PSA: Probabilistic sensitivity analysis
PTT: Partial thromboplastin time
PUFA: Polyunsaturated fatty acid
RR: Relative risk
TNF: Tumor necrosis factor
TSA: Trial sequential analysis
UK: United Kingdom
US: United States
USD: US dollars
ω-3: Omega-3
ω-6: Omega-6
