# Supplementary material for: Omega-3 fatty acid-containing parenteral nutrition in ICU patients: systematic review with meta-analysis and cost-effectiveness analysis
Source: Crit Care. 2020 Nov 3;24:634. doi: 10.1186/s13054-020-03356-w (PMC7607851; doi:10.1186/s13054-020-03356-w)
Supplement: Supplementary file 1 — Additional file 1: Complete input parameters, included studies details and additional results and analyses. [file 13054_2020_3356_MOESM1_ESM.docx]

**Tables:**

**Supplementary Table 1.** Model inputs: Clinical and economic parameters as well as overall efficacy estimates.

| **Model inputs** | **France** | **Germany** | **Italy** | **Spain** | **UK*** | **US*** |
| --- | --- | --- | --- | --- | --- | --- |
| **Clinical input parameters** | | | | | | |
| Mean HLOS, days | 31.2 ± 18.5 (1) | 29.1 ± 18.7 (2) | 36.8 ± 28.5 (3) | 45.85 ± 23.9 (4) | 19.65 ± 19.3 (5) | 20.14 ± 15.5 (6) |
| Infection, % | 47 (1) | 19 (7) | 45 (8) | 46 (4) | 19 (5) | 34 (6) |
| Mean PN duration ± SD, days | 14 ± 8 (1) | 9 ± 5 (2) | 7 ± 6 (9) | 19 ± 15 (4) | 9 ± 5 (5) | 7 ± 6 (6) |
| Mortality, % | 28 (1) | 36 (2) | 15 (3) | 46 (4) | 36 (5) | 21 (6) |
| **Economic input parameters** | | | | | | |
| Mean cost/day, € | 1,136 (10) | 1,556 (11) | 1,108 (12) | 981 (13) | 1,808 (14) | 2,906 (6) |
| Mean cost of infection, € | 1,162 (15) | 2,006 (16) | 1,855 (17) | 2,085 (18) | 825 (19) | 6,625 (20) |
| Mean cost of PN/day, € | 28^†^ | 117^†^ | 96^†^ | 14^†^ | 66^†^ | 12^†^ |
| Mean cost of ω-3 FA-containing PN/day, € | 26^†^ | 130^†^ | 154^†^ | 22^†^ | 73^†^ | 23^†^ |
| **ω-3 FA-containing PN efficacy,** pooled estimate ± SD | **HLOS mean difference, days** | | | **Infection relative risk** | | |
|  | -3.05 ± 1.001 | | | 0.62 ± 0.12 | | |

*GBP and USD converted to EUR using the average exchange rates valid in July 2019: GBP-EUR: 1.11224; USD-EUR: 0.89825.

^#^Data combined from two groups according to the Cochrane handbook.(21)

^†^Fresenius Kabi data on file.

EUR, euro; FA, fatty acid; GBP, pound sterling; HLOS, hospital length of stay; ω-3, omega-3; PN, parenteral nutrition; SD, standard deviation; USD, US dollar.

**Supplementary Table 2.** Characteristics of included randomized controlled trials (n=24).

| **Study** | **Setting (n*)** | **ω-3 FA containing lipid emulsion** | **Standard lipid emulsion** | **Clinical outcomes** | **Laboratory outcomes** |
| --- | --- | --- | --- | --- | --- |
| Antebi et al., 2004 | Major surgery (n=20) | SO/MCT/OO/FO | SO | – | ALT, AST, CRP, α-T, TG, GGT |
| Barbosa et al., 2010 | SIRS or sepsis (n=23, received study treatments) | SO/MCT/FO | SO/MCT | Mortality, HLOS, ICU LOS, OI | AA, ALT, AST, bilirubin, CRP, DHA, EPA, GTT, IL-6, LTB4, OI, PTT, Lac, Plt, TNF |
| Berger et al., 2008 | Abdominal aortic aneurism surgery (n=24, completed trial) | SO/MCT/FO | SO/MCT | Mortality, HLOS, ICU LOS, Ventilation days | Α-T, AA, CRP, DHA, EPA, TG |
| Chen et al., 2017 | Severe sepsis with grade III acute gastrointestinal injury (n=78) | SO/FO | SO | Mortality | CRP |
| Chen et al., 2017 | Patients with septicemia and intestinal dysfunction (n=48) | Standard TPN/FO | Standard TPN | ICU LOS, Mortality | CRP |
| Donoghue V et al., 2018 | SIRS or sepsis and/or ARDS (n=75) | SO/OO/FO | SO/OO | ICU LOS, Ventilation days, OI | ALT, AST, GTT, bilirubin, CRP, IL-6, TG, TNF, DHA, EPA, AA, α-T |
| Friesecke et al., 2008 | Critically ill medical (n=165) | SO/MCT/FO | SO/MCT | Mortality, infections, HLOS, ICU LOS, Bleeding events, Ventilation days | IL-6, TBU |
| Grau-Carmona 2015 | Medical and surgical ICU patients (n=175) | SO/MCT/FO | SO/MCT | Mortality, infections, HLOS, ICU LOS, Ventilation days | – |
| Gultekin et al., 2014 | ICU patients with sepsis (n=32) | SO/OO/FO | SO/OO | Mortality, HLOS | CRP, IL-6, LTB4, TG, TNF |
| Han et al., 2012 | Major surgery (n=38) | SO/MCT/FO | SO/MCT | Infections | IL-6, TNF |
| Heller et al., 2002 | Cancer, major abdominal surgery (n=44) | SO/FO | SO | – | Plt, PT, PTT |
| Heller et al., 2004 | Cancer, major abdominal surgery (n=44) | SO/FO | SO | HLOS, ICU LOS | ALT, AST, bilirubin, CRP |
| Morlion et al., 1996 | Major abdominal surgery (n=20) | SO/FO | SO | – | AA, EPA, DHA, LTB4, LTB5 |
| Piper et al., 2009 | Major abdominal or craniomaxillofacial surgery (n=44) | SO/MCT/OO/FO | SO/OO | Ventilation days | ALT, AST, Plt, TG |
| Roulet et al., 1997 | Cancer, esophagectomy (n=19, completed trial) | SO/FO | SO | – | AA, DHA, EPA, BT |
| Sabater et al., 2011 | ARDS (n=16) | SO/MCT/FO | SO | Mortality | LTB4 |
| Stephenson et al., 2013 | Surgery for hepatic colorectal metastasis (n=20) | SO/MCT/FO | SO/MCT | – | AA, DHA, EPA |
| Wachtler et al., 1997 | Cancer, major intestinal surgery (n=40) | SO/MCT/FO | SO/MCT | Infections, HLOS, ICU LOS | IL-6, LTB4, LTB5, LTB ratio, TNF |
| Wang et al., 2008 | Severe acute pancreatitis (n=40) | SO/FO | SO | Mortality, infections, HLOS, ICU LOS, OI | CRP, EPA, IL-6, OI |
| Wang et al., 2009 | Severe acute pancreatitis (n=56) | SO/FO | SO | Mortality, infections | – |
| Weiss et al., 2002 | Gastrointestinal surgery (n=24) | SO/FO | SO | Mortality, infections, HLOS, ICU LOS | IL-6, TNF |
| Wendel et al., 2007 | Cancer, major abdominal surgery (n=44) | SO/FO | SO | – | TG |
| Wichmann et al., 2007 | Major intestinal surgery (n=256) | SO/MCT/FO | SO | Mortality, infections, HLOS, ICU LOS | Α-T, AST, bilirubin, Cr, CRP, EPA, GGT, LTB5, LTB ratio, Plt, PT, TG |
| Zhu et al., 2013 | Pancreaticoduodenectomy (n=76) | SO/MCT/FO | SO/MCT | Mortality, infection, HLOS, hospital readmission, Ventilation days | ALT, AST, bilirubin |

*Number of patients randomized was listed if available, but if not available an alternative descriptor was used for the patient population/number.

AA, (%) content of arachidonic acid in serum/cellular membranes; α-T, α-tocopherol; ALT, alanine aminotransferase; ARDS, acute respiratory distress syndrome; AST, aspartate aminotransferase; Cr, serum creatinine; CRP, C-reactive protein; DHA, (%) docosahexaenoic acid content in serum/cellular membranes; EPA, (%) eicosapentaenoic acid content in serum/cellular membranes; FA, fatty acid; FO, fish oil emulsion; GGT, gamma-glutamyl transferase; HLOS, hospital length of stay; ICU, intensive care unit; IL, interleukin; Lac, lactate; LOS, length of stay; LTB, leukotriene B; LTB ratio, LTB5:LTB4; MCT, medium-chain triglycerides; ω-3, omega-3; OI, oxygenation index; OO, olive oil emulsion; Plt, Platelet; PT, prothrombin time; PTT, partial thromboplastin time; SIRS, systemic inflammatory response syndrome; SO, soybean oil emulsion; TBU, transfused blood unit; TG, triglycerides; TNF, tumor necrosis factor.

**Supplementary Table 3.** Summary of laboratory outcomes comparing ω-3 FA-containing and standard PN.

| **Outcome/subgroup** | **Number of studies** | **Number of patients** | **Effect measure (statistical method)** | **Effect estimate (95% CI) (22)** |
| --- | --- | --- | --- | --- |
| Oxygenation index | 3 | 129 | MD (IV, FE) | -29.12 [-96.10, 37.85] |
| AST level | 6 | 456 | MD (IV, RE) | -7.41 [-17.83, 3.01] |
| **ALT level** | **5** | **197** | **MD (IV, RE)** | **-11.19 [-21.71, -0.67]** |
| GGT level | 4 | 365 | MD (IV, FE) | -11.57 [-34.24, 11.10] |
| Bilirubin (total) | 5 | 455 | MD (IV, RE) | -4.81 [-9.86, 0.24] |
| Triglyceride level |  |  | MD (IV, RE) |  |
| **Α-tocopherol** | **3** | **148** | **MD (IV, RE)** | **16.77 [9.13, 24.40]** |
| **EPA** | **5** | **209** | **St.MD (IV, RE)** | **3.58 [1.48, 5.67]** |
| **DHA** | **3** | **109** | **St.MD (IV, RE)** | **2.62 [0.60, 4.64]** |
| AA | 3 | 109 | St.MD (IV, RE) | 0.00 [-0.37, 0.38] |
| LBT5 | 3 | 316 | St.MD (IV, RE) | 2.08 [-0.79, 4.95] |
| LBT4 | 6 | 176 | MD (IV, RE) | -1.11 [-22.40, 20.18] |
| **LBT5/LBT4** | **2** | **100** | **MD (IV, RE)** | **0.07 [0.06, 0.07]** |
| CRP | 8 | 529 | MD (IV, RE) | -8.66 [-25.38, 8.05] |
| **IL-6** | **7** | **399** | **MD (IV, RE)** | **-40.58 [-55.93, -25.24]** |
| Platelet | 3 | 324 | MD (IV, FE) | 12.89 [-14.74, 40.52] |
| **TNF-α** | **5** | **201** | **MD (IV, RE)** | **-35.22 [-58.65, -11.79]** |
| Transfused blood unit | 2 | 209 | MD (IV, FE) | -0.38 [-2.54, 1.77] |

Statistically significant parameters in bold.

AA, arachidonic acid; ALT, alanine aminotransferase; AST, aspartate aminotransferase ; CI, confidence interval; CRP, C-reactive-protein; DHA, docosahexaenoic acid; EPA, eicosapentaenoic acid; FE, fixed effects; GGT, γ-glutamyl transferase; IL, interleukin; IV, inverse variance; LOS, length of stay; LT, leukotriene; MD, mean difference; M–H, Mantel-Haenszel; n, number; PT, prothrombin time; PPT, partial thromboplastin time; RE, random effects; RR, relative risk; St.MD, standardized mean difference; TBU, transfused blood units; TNF, tumor necrosis factor.

**Supplementary Table 4.** Summary of confidence in cumulative estimates for clinical outcomes.

| **Certainty assessment** | | | | | | | **№ of patients** | | **Effect** | | **Certainty** |
| --- | --- | --- | --- | --- | --- | --- | --- | --- | --- | --- | --- |
| **№ of studies** | **Study design** | **Risk of bias** | **Inconsistency** | **Indirectness** | **Imprecision** | **Other considerations** | **Main analysis** | **Placebo** | **Relative (95% CI)** | **Absolute (95% CI)** |  |
| **Infection rate** | | | | | | | | | | | |
| 8 | Randomized trials | Not serious | Not serious | Not serious | Not serious | None | 49/401 (12.2%) | 75/394 (19.0%) | **RR 0.62** (0.45 - 0.86) | **72 fewer per 1.000** (27 - 105) | **⨁⨁⨁⨁ High** |
| **Hospital length of stay** | | | | | | | | | | | |
| 11 | Randomized trials | Not serious | **Serious^a^** | Not serious | Not serious | None | 440 | 432 | – | **MD 3.05 lower** (5.03 - 1.07) | **⨁⨁⨁◯ Moderate** |
| **ICU length of stay** | | | | | | | | | | | |
| 11 | Randomized trials | Not serious^b^ | **Serious^a^** | Not serious | Not serious | None | 450 | 440 | – | MD **1.89 lower** (3.33 -0.45) | **⨁⨁⨁◯ Moderate** |

^a^Some inconsistency exists due to statistically significant heterogeneity.

^b^The proportion of information from studies at high risk is insufficient to affect the interpretation of the result.

CI, confidence interval; MD, mean difference; RR, relative risk.

**Figures:**

**Supplementary Figure 1.** Simplified model structure.

**
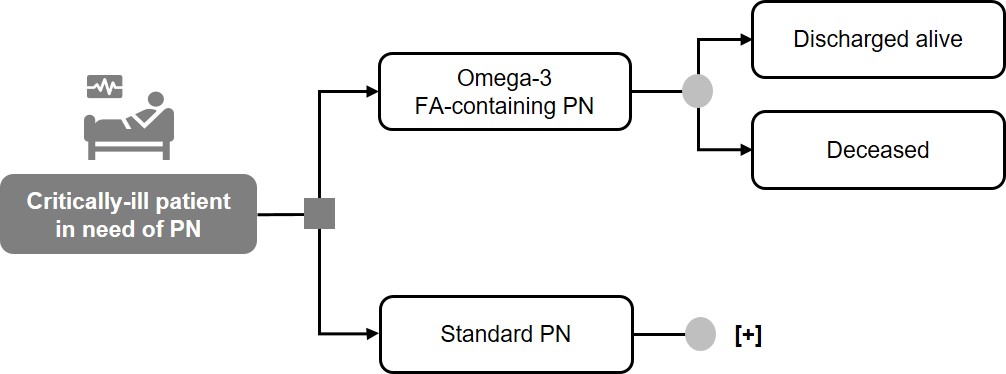
**

FA, fatty acid; PN, parenteral nutrition.

**Supplementary Figure 2.** Studies selection flow diagram and main reasons for study exclusion.

**
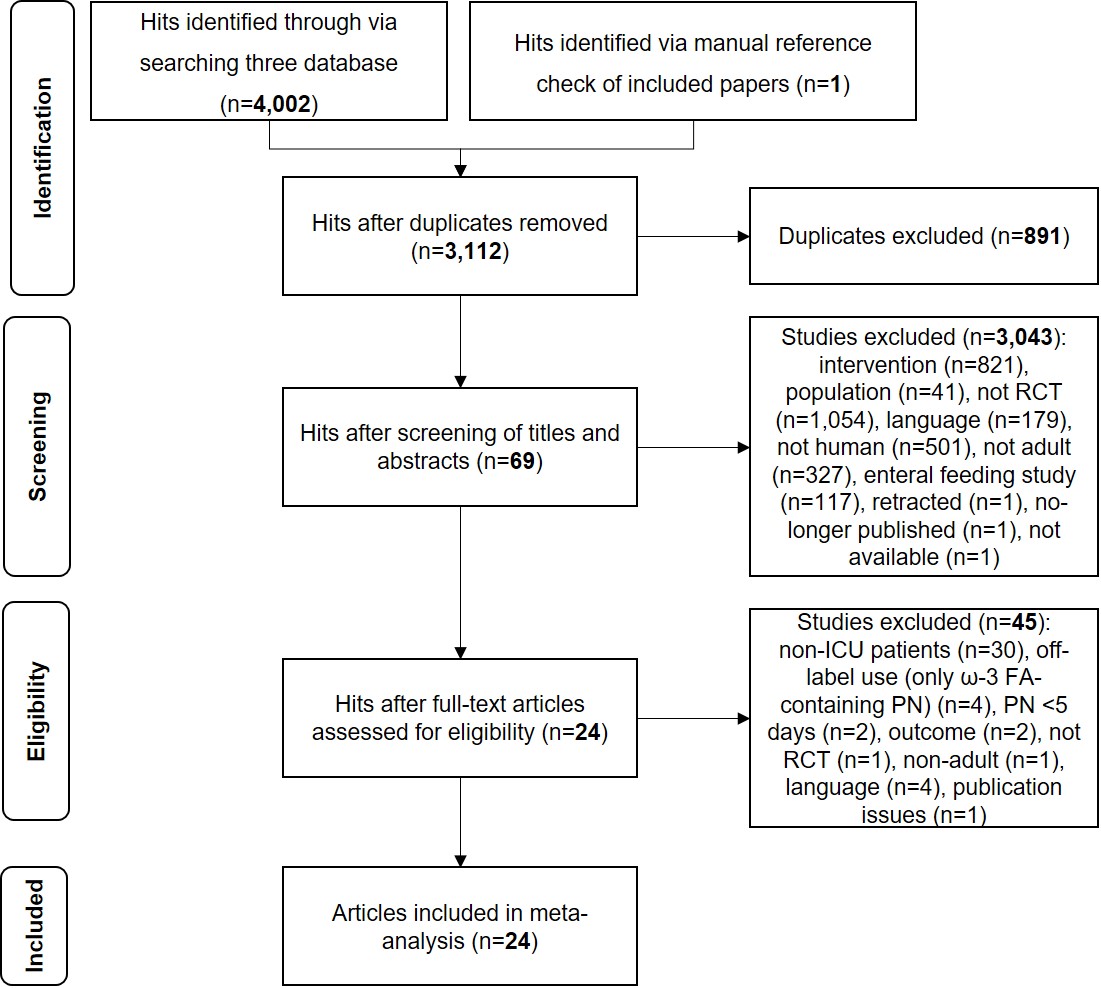
**

FA, fatty acid; ICU, intensive care unit; ω-3, omega-3; PN, parenteral nutrition; RCT, randomized controlled trial.

**Supplementary Figure 3.** TSA of significant clinical outcomes a) infection rate, b) HLOS and c) ICU LOS.


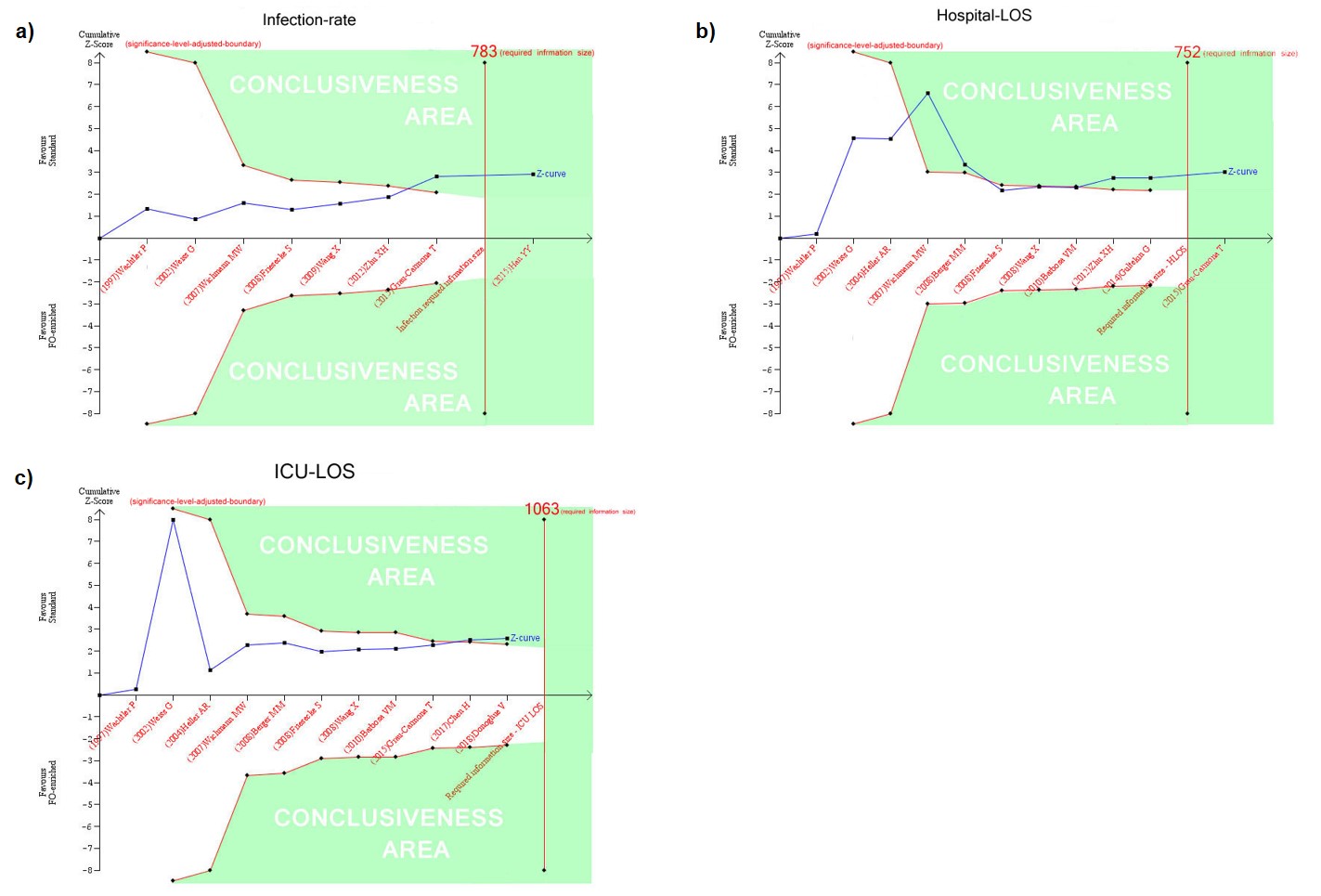


The red inward-sloping lines represent the trial sequential monitoring boundaries, with dots highlighting alphas adjusted at each trial sequentially entering the analysis. The blue line represents the evolution of the cumulative Z-score. If the cumulative Z-curve crosses the red monitoring boundaries, and the analyzed sample exceeds the required information size, the conclusiveness of the results is confirmed. Hence the results for infection rate and ICU LOS are conclusive. Although the required information size has not been reached for HLOS, the cumulative Z-curve crossing the monitoring boundaries also confirms the conclusiveness.

HLOS, hospital length of stay; ICU, intensive care unit; LOS, length of stay; TSA, trial sequential analysis.

**Supplementary Figure 4.** Tornado plots comparing standard PN and ω-3 FA-containing PN.


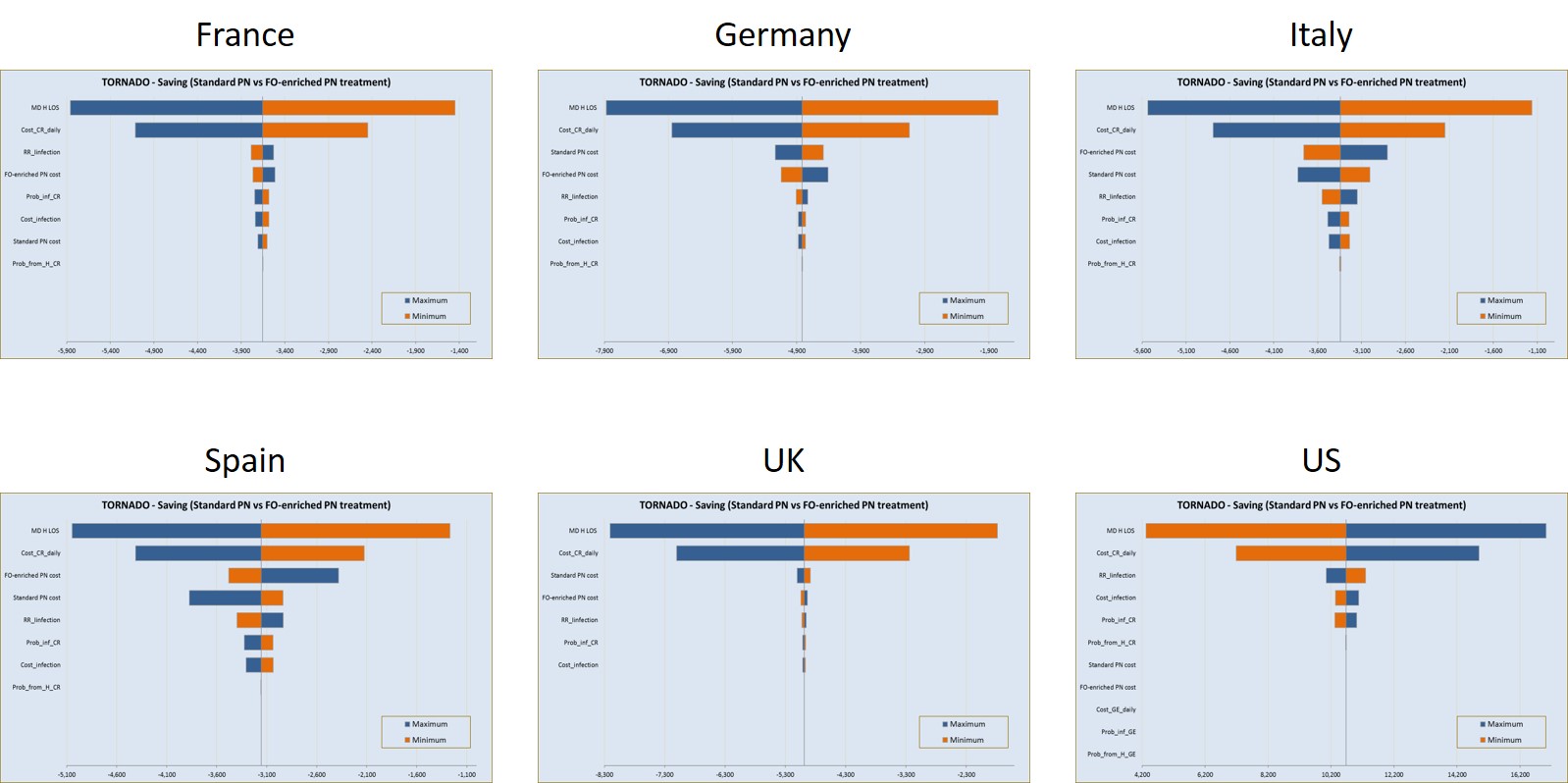
 Parameters ranked by degree of influence (most influential parameters at the top).

CR, critically ill; FA, fatty acid; HLOS, hospital length of stay; ω-3, omega-3; MD, mean difference; PN, parenteral nutrition; Prob from H, probability to be discharged alive; RR, relative risk.

**Supplementary Figure 5.** Begg’s funnel plots of clinical outcomes with pseudo 95% confidence intervals.


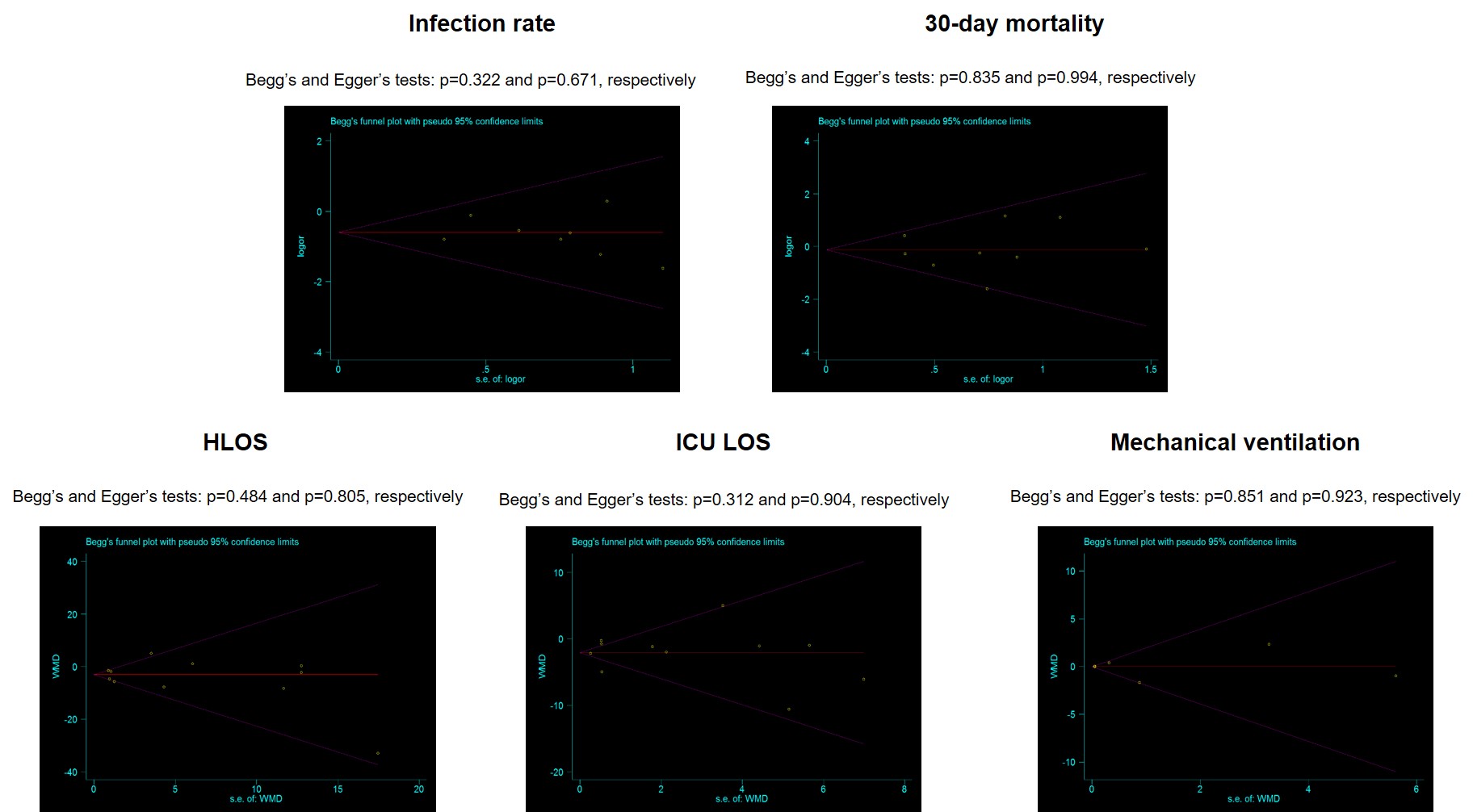


OR, odds ratio; SE, standard error; WMD, weighted mean difference.

**References**

1. Bauer P, Charpentier C, Bouchet C, Nace L, Raffy F, Gaconnet N. Parenteral with enteral nutrition in the critically ill. Intensive Care Med. 2000;26(7):893-900.

2. Heller AR, Rossler S, Litz RJ, Stehr SN, Heller SC, Koch R, et al. Omega-3 fatty acids improve the diagnosis-related clinical outcome. Crit Care Med. 2006;34(4):972-9.

3. Radrizzani D, Bertolini G, Facchini R, Simini B, Bruzzone P, Zanforlin G, et al. Early enteral immunonutrition vs. parenteral nutrition in critically ill patients without severe sepsis: a randomized clinical trial. Intensive Care Med. 2006;32(8):1191-8.

4. Mateu-de Antonio J, Grau S, Luque S, Marin-Casino M, Albert I, Ribes E. Comparative effects of olive oil-based and soyabean oil-based emulsions on infection rate and leucocyte count in critically ill patients receiving parenteral nutrition. Br J Nutr. 2008;99(4):846-54.

5. Harvey SE, Parrott F, Harrison DA, Sadique MZ, Grieve RD, Canter RR, et al. A multicentre, randomised controlled trial comparing the clinical effectiveness and cost-effectiveness of early nutritional support via the parenteral versus the enteral route in critically ill patients (CALORIES). Health Technol Assess. 2016;20(28):1-144.

6. Magee G, Zaloga GP, Turpin RS, Sanon M. A retrospective, observational study of patient outcomes for critically ill patients receiving parenteral nutrition. Value Health. 2014;17(4):328-33.

7. Deutsche Nationale Punkt-Prävalenzstudie zu nosokomialen Infektionen und Antibiotika-Anwendung 2011;<https://www.nrz-hygiene.de/fileadmin/nrz/download/PPS-Abschlussbericht-Stand05-08-2013final.pdf>

8. Luzzati R, Antozzi L, Bellocco R, Del Bravo P, Mirandola M, Procaccio F, et al. Prevalence of nosocomial infections in intensive care units in Triveneto area, Italy. Minerva Anestesiol. 2001;67(9):647-52.

9. Progetto Margherita. ICU report 2010;<http://www.giviti.marionegri.it/MargheritaDue.asp>.

10. Parienti JJ, Lucet JC, Lefort A, Armand-Lefevre L, Wolff M, Caron F, et al. Empirical therapies among adults hospitalized for community-acquired upper urinary tract infections: A decision-tree analysis of mortality, costs, and resistance. Am J Infect Control. 2015;43(9):e53-9.

11. Arefian H, Hagel S, Heublein S, Rissner F, Scherag A, Brunkhorst FM, et al. Extra length of stay and costs because of health care-associated infections at a German university hospital. Am J Infect Control. 2016;44(2):160-6.

12. Tan SS, Bakker J, Hoogendoorn ME, Kapila A, Martin J, Pezzi A, et al. Direct cost analysis of intensive care unit stay in four European countries: applying a standardized costing methodology. Value Health. 2012;15(1):81-6.

13. Rello J, Nieto M, Sole-Violan J, Wan Y, Gao X, Solem CT, et al. Nosocomial pneumonia caused by methicillin-resistant Staphylococcus aureus treated with linezolid or vancomycin: A secondary economic analysis of resource use from a Spanish perspective. Med Intensiva. 2016;40(8):474-82.

14. Marti J, Hall P, Hamilton P, Lamb S, McCabe C, Lall R, et al. One-year resource utilisation, costs and quality of life in patients with acute respiratory distress syndrome (ARDS): secondary analysis of a randomised controlled trial. J Intensive Care. 2016;4:56.

15. Defez C, Fabbro-Peray P, Cazaban M, Boudemaghe T, Sotto A, Daures JP. Additional direct medical costs of nosocomial infections: an estimation from a cohort of patients in a French university hospital. J Hosp Infect. 2008;68(2):130-6.

16. Leistner R, Gurntke S, Sakellariou C, Denkel LA, Bloch A, Gastmeier P, et al. Bloodstream infection due to extended-spectrum beta-lactamase (ESBL)-positive K. pneumoniae and E. coli: an analysis of the disease burden in a large cohort. Infection. 2014;42(6):991-7.

17. Tarricone R, Torbica A, Franzetti F, Rosenthal VD. Hospital costs of central line-associated bloodstream infections and cost-effectiveness of closed vs. open infusion containers. The case of Intensive Care Units in Italy. Cost Eff Resour Alloc. 2010;8:8.

18. Cots F, Riu M, Pi-Sunyer T, Terradas R, Grau S, Castells X. Incremental cost due to nosocomial infections.<http://www.postermedic.com/parcdesalutmar/npimas072809/pdfbaja/npimas072809.pdf>.

19. Plowman R, Graves N, Griffin MA, Roberts JA, Swan AV, Cookson B, et al. The rate and cost of hospital-acquired infections occurring in patients admitted to selected specialties of a district general hospital in England and the national burden imposed. J Hosp Infect. 2001;47(3):198-209.

20. Butler AM, Olsen MA, Merz LR, Guth RM, Woeltje KF, Camins BC, et al. Attributable costs of enterococcal bloodstream infections in a nonsurgical hospital cohort. Infect Control Hosp Epidemiol. 2010;31(1):28-35.

21. Higgins JPT, Green S. Cochrane handbook for systematic reviews of interventions. Version 5.1.0 [updated March 2011]. The Cochrane Collaboration. 2011;p. Table 7.7.a: Formulae for combining groups.

22. Levy MM, Fink MP, Marshall JC, Abraham E, Angus D, Cook D, et al. 2001 SCCM/ESICM/ACCP/ATS/SIS International Sepsis Definitions Conference. Crit Care Med. 2003;31(4):1250-6.
